# Feasibility and Safety of Cytosorb Application in Patients With Acute Liver Failure

**DOI:** 10.1111/liv.70420

**Published:** 2025-11-14

**Authors:** Haselwanter Patrick, Fairfield Seanna, Reinhold Riva, Riedl‐Wewalka Marlene, Schmid Monika, Balcar Lorenz, Stättermayer Albert Friedrich, Reiberger Thomas, Trauner Michael, Zauner Christian, Schneeweiss‐Gleixner Mathias

**Affiliations:** ^1^ Department of Medicine III Division of Gastroenterology and Hepatology, Intensive Care Unit 13H1, Medical University of Vienna Vienna Austria; ^2^ Clinical Research Group MOTION, Medical University of Vienna Vienna Austria; ^3^ Christian‐Doppler Laboratory for Portal Hypertension and Liver Fibrosis, Medical University of Vienna Vienna Austria

## Abstract

**Background and Aims:**

Acute liver failure (ALF) is a rare, life‐threatening condition with high mortality and limited therapeutic options beyond liver transplantation. Cytosorb adsorber can be easily applied to continuous renal replacement therapy (CRRT) and potentially support liver function by removing bilirubin and cytokines.

**Methods:**

This retrospective single‐centre study included all ALF patients admitted to the intensive care unit (ICU) at the Division of Gastroenterology and Hepatology of the Vienna General Hospital from 2012 to 2025 receiving CRRT.

**Results:**

Twenty‐eight ALF patients (median SOFA 11, SAPS II 43) were included, with high incidences of vasopressor use (82.1%) and invasive ventilation (85.7%). Fourteen received adjunctive Cytosorb therapy. Complications (hemodynamic instability, citrate accumulation, bleeding, hypofibrinogenemia, thrombocytopenia) were similar in patients with or without Cytosorb. Bilirubin levels significantly decreased after Cytosorb initiation (from 20.85 to 12.32 mg/dL at 24 h, and to 9.46 mg/dL after treatment; *p* < 0.001), GGT, ALAT, and platelets also declined post‐treatment. Cytosorb patients had lower SAPS II scores (median: 38.5 vs. 45.5, *p* = 0.039), were more often listed for HU‐LTx (92.9% vs. 42.9%, *p* = 0.005), and had higher transplantation rates (64.3% vs. 28.6%, *p* = 0.058). Therefore, ICU survival (71.4% vs. 42.9%; *p* = 0.127) and 6‐month survival (71.4% vs. 28.6%; *p* = 0.023) were higher in the Cytosorb group.

**Conclusions:**

Cytosorb appeared to be a feasible and well‐tolerated blood purification tool with potential beneficial effects in ALF patients. Improved survival with Cytosorb likely reflects confounding by patient selection and higher HU‐LTx rates. Prospective studies are warranted to clarify the clinical impact of adjunctive Cytosorb therapy in ALF patients.

**Summary**

- Acute liver failure (ALF) is a rare but severe condition occurring in individuals without—by definition—pre‐existing chronic liver disease that remains without effective treatment options except for liver transplantation.
- Cytosorb is an easily‐applicable blood purification tool installed into conventional dialysis with the aim to remove circulating toxins and proinflammatory cytokines.
- Cytosorb seemed to be a safe blood purification tool for ALF patients, improved liver biochemistry and hemodynamics and was without safety signals when applied in ALF patients with an indication for haemodialysis.

**Abbreviations**
ACLF: acute‐on‐chronic liver failure
AIH: autoimmune hepatitis
ALAT: alanine aminotransferase
ALF: acute liver failure
ALI: acute liver injury
AP: alkaline phosphatase
ASAT: aspartate aminotransferase
CRP: C‐reactive protein
CRRT: continuous renal replacement therapy
CVVHD: continuous venovenous haemodialysis
DILI: drug‐induced liver injury
EASL: European Association for the Study of the Liver
GGT: gamma‐glutamyl transferase
HE: hepatic encephalopathy
HU‐LTx: high‐urgency liver transplantation
HVPE: high‐volume plasma exchange
ICU: intensive care unit
IL: interleukin
IMV: invasive mechanical ventilation
INR: international normalised ratio
IQR: interquartile range
LOS: length of stay
PCT: procalcitonin
SAPS II: Simplified Acute Physiology Score II
SOFA: Sequential Organ Failure Assessment
TNF: tumour necrosis factor
WBC: white blood cell count

## 1 Introduction

Acute liver failure (ALF) is a rare but life‐threatening disease among previously liver‐healthy patients characterised by severe hepatic injury and, consequently, loss of liver function. Patients with ALF require immediate management at specialised (liver) intensive care units (ICU) and prompt evaluation for high‐urgency liver transplantation (HU‐LTx) [1, 2]. Systemic inflammation, reflected by elevated levels of proinflammatory cytokines (e.g., interleukin [IL]‐6 and tumour necrosis factor [TNF]‐α), plays a central role in the pathogenesis and progression of ALF, contributing to multi‐organ failure and dismal prognosis. Consequently, targeted modulation of systemic inflammation through extracorporeal cytokine removal has emerged as a pivotal therapeutic strategy in ALF, potentially supporting liver function while bridging critically ill patients to HU‐LTx or liver regeneration [3, 4, 5].

Over the past decades, a variety of artificial and bioartificial liver‐assist devices have been developed to provide temporary hepatic support, serving as a bridging device to transplantation or recovery. These devices either consist of selective membranes with various pore sizes and adsorbent affinities to remove water‐soluble and albumin‐bound toxins (e.g., MARS, OPAL, Prometheus) or cartridges containing liver cells to support detoxification and liver synthesis (e.g., ELAD) [6, 7, 8, 9, 10]. Despite encouraging outcomes in smaller trials and case series, including improvements in transplant‐free survival and hemodynamic stability, none of these devices demonstrated a significant clinical benefit in larger randomised‐controlled trials [1, 3, 7, 11]. In addition, concerns were raised about side effects like thrombocytopenia and circuit thrombosis [3]. Most importantly, these devices are highly complex, resource‐intensive, and require specialised expertise, thus limiting their use to tertiary care centres [3, 6, 10]. High‐volume plasma exchange (HVPE) is the only adjunctive therapy currently recommended in ALF, based on evidence suggesting improved transplant‐free survival in one prospective clinical trial. Notably, this effect was most pronounced in patients not listed for HU‐LTx due to contraindications or comorbidities [12].

Cytosorb is a hemadsorbtive cartridge that can be easily applied in addition to continuous renal replacement therapy (CRRT), potentially supporting liver function by removal of endogenous toxins (e.g., bilirubin) and excessive proinflammatory cytokines. Cytosorb has already been investigated in patients with acute‐on‐chronic liver failure (ACLF), where it significantly reduced bilirubin, procalcitonin, and IL‐6 levels. Importantly, adding Cytosorb seemed to be a feasible and safe approach in ACLF patients. Its clinical benefit, particularly in terms of survival, still remains unclear due to the lack of larger prospective studies in this patient population [13]. Data on Cytosorb use in ALF patients is scarce and limited to a few case reports [14, 15, 16, 17]. In a small propensity‐matched retrospective study comparing ALF/ACLF patients receiving either MARS or Cytosorb, laboratory parameters significantly improved in the Cytosorb group. However, no significant impact on survival and ICU length of stay (LOS) was observed [15].

Given the ongoing lack of effective supportive therapies for patients with ALF, we aimed to identify potential complications associated with Cytosorb use and to assess its impact on hepatic and extrahepatic organ function in critically ill patients with ALF.

## 2 Patients, Material, and Methods

### 2.1 Study Design and Setting

We conducted a retrospective, observational, single‐centre study including patients with ALF admitted to the ICU of the Department of Gastroenterology and Hepatology from January 1, 2012, to January 1, 2025. All adult patients (18 years and older) with ALF receiving treatment with conventional CRRT were included in this study, as depicted in Figure S1. Diagnosis of ALF was defined according to the European Association for the Study of the Liver (EASL) guidelines [1]. Depending on the development from jaundice to hepatic encephalopathy (HE), patients were differentiated into hyperacute (0–7 days), acute (8–28 days), or subacute (28 days–12 weeks) liver failure [18]. Patients were followed at ICU admission (baseline) until ICU discharge, HU‐LTx, or death. We focused on (i) safety and complication rates of CRRT±Cytosorb, and (ii) the effects of Cytosorb therapy on laboratory parameters and outcome.

The study was approved by the local ethics committee of the Medical University of Vienna (1229/2024). Due to the retrospective study design, no informed consent was required. This study was conducted in accordance with the principles of Good Clinical Practice, as outlined by the European Commission and the revised version of the Declaration of Helsinki [19].

### 2.2 CRRT and Cytosorb Therapy

All included ALF patients had an indication (i.e., hyperammonemia, acute kidney failure, or acid–base disturbances) for haemodialysis. CRRT was routinely conducted as continuous venovenous haemodialysis (CVVHD; MultiFiltrate, Fresenius Medical Care). The decision to initiate additional Cytosorb adsorber therapy was made at the discretion of the treating physicians. Cytosorb therapy was always administered in conjunction with standard intensive care treatment, as per the current EASL guidelines [1].

Cytosorb adsorber was used in the pre‐filter position, and adsorbers were changed after 8, 16, or 24 h, as decided by the treating physicians. Regional citrate anticoagulation was primarily conducted during extracorporeal blood circulation. In the case of citrate accumulation, the anticoagulation was changed to antithrombin III, heparin, or CRRT without anticoagulation. Citrate accumulation was rigorously monitored, with the total calcium to ionised calcium ratio checked at least three times daily [20].

### 2.3 Parameters of Interest

While baseline parameters were collected at ICU admission, we additionally investigated patients directly before Cytosorb, 1 day after initiation, and after discontinuation to explore the impact on laboratory, hemodynamic, and outcome parameters. Relevant laboratory parameters included liver chemistry [bilirubin, aspartate aminotransferase (ASAT), alanine aminotransferase (ALAT), alkaline phosphatase (AP), gamma‐glutamyl transferase (GGT), and ammonia], inflammation parameters (C‐reactive protein [CRP], procalcitonin [PCT], IL‐6), coagulation parameters (fibrinogen, international normalised ratio [INR]), white blood count (WBC) and platelets. In the control group (CRRT only), we collected data at the same time points as reference values.

### 2.4 Adverse Events

To analyse the safety of Cytosorb application in ALF patients, we focused on known complications associated with extracorporeal circulation, both with and without Cytosorb, including hemodynamic instability (vasopressor support or mean blood pressure < 65 mmHg at Cytosorb/CRRT discontinuation), hypofibrinogenemia (fibrinogen levels < 100 mg/dL at Cytosorb/CRRT discontinuation), severe thrombocytopenia (platelets below < 50 G/L at Cytosorb/CRRT discontinuation), citrate accumulation (total calcium to ionised calcium ratio > 2.5 or evident signs of citrate accumulation) and major bleeding (fatal bleedings, bleeding with a decrease in haemoglobin level of ≥ 2 g/dL or bleeding leading to transfusion of ≥ 2 units of packed red blood cells during Cytosorb/CRRT treatment) [21].

### 2.5 Data Collection

Data was prospectively recorded in electronic patient charts (IntelliSpace Critical Care and Anaesthesia, Philips, Amsterdam, Netherlands) for routine documentation. For this study, the following data was collected retrospectively from these electronic patient charts: basic characteristics (sex, age, height, weight, body mass index, vital signs, and comorbidities), laboratory values, safety events, ICU‐specific parameters (medication, nutrition, and extracorporeal life support), and outcome parameters. We particularly focused on data collection for extracorporeal therapy, including daily records on CRRT (i.e., admission to CRRT, duration of CRRT, citrate accumulation, and bleeding events) and Cytosorb application (i.e., admission to Cytosorb, duration of Cytosorb, and adsorber changes). Disease severity scores, such as the Sequential Organ Failure Assessment score (SOFA) and the Simplified Acute Physiology Score (SAPS II) score, were calculated 24 h after ICU admission [22, 23].

### 2.6 Statistical Analysis

Data were presented as medians with interquartile ranges (IQRs). Qualitative parameters are presented in absolute numbers, along with relative proportions expressed as percentages. To compare the Cytosorb group and control group, the Chi‐Square Test was used for categorical variables, and the Mann–Whitney *U*‐test was used as a nonparametric test for metric variables. Statistical significance was defined as *p* < 0.05, without consideration of multiple comparisons. To compare median changes of paired laboratory parameters, we used the paired Wilcoxon test. The statistical analyses were performed with GraphPad Prism 10 (GraphPad Software, CA, USA) and IBM SPSS Statistics 28 (IBM, New York, NY, USA).

## 3 Results

### 3.1 Patient Cohort and Clinical Characteristics

**FIGURE 1:**
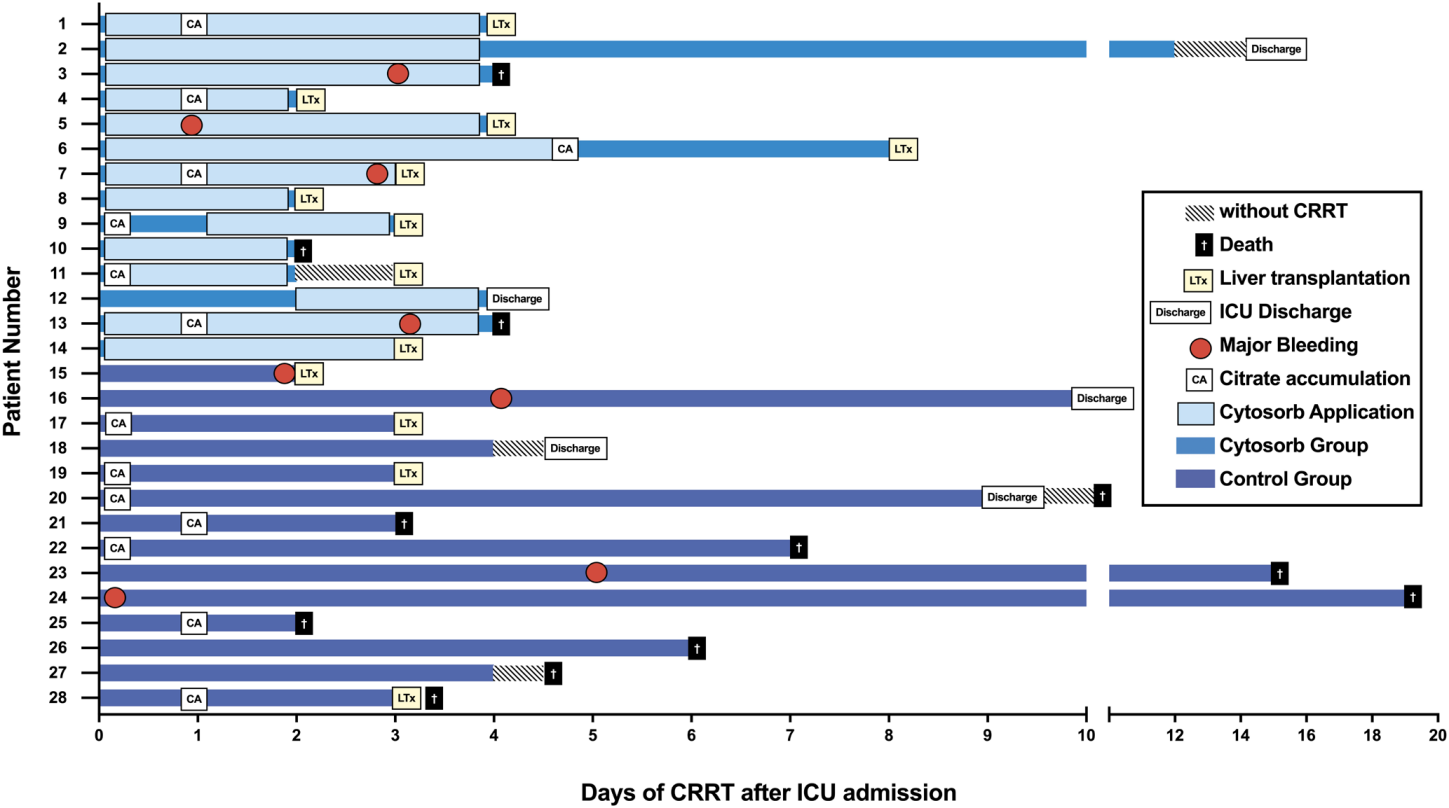
Timeline of Cytosorb and control group receiving CRRT. Individual patient courses refer to the numbers in Table 2. Patients receiving HU‐LTx were highlighted with “LTx” in a yellow square. ICU discharge is marked with a “Discharge” in a white square, whereas patients' death is marked with a cross in a black square. Individual CRRT time was given in bars with light blue in the Cytosorb + CRRT group and dark blue in the CRRT group. If patients died, were discharged, or received HU‐LTx short after CRRT, time was given in bars with oblique black stripes. Citrate accumulation was marked as CA in a white square, and a major bleeding event during CRRT was marked as a red dot. CA, citrate accumulation; CRRT, continuous renal replacement therapy; ICU, intensive care unit; LTx, liver transplantation.

Overall, 28 patients with CRRT and ALF were included. Of those, 14 patients received Cytosorb add‐on to CRRT during the study period. The baseline and individual characteristics of all patients are presented in Table 1 and Table 2, while their clinical course is depicted in Figure 1. The median age was 47.1 (IQR: 32.5–56.4) years. The most common underlying etiologies of ALF were autoimmune hepatitis (AIH, *n* = 7, 25%) and viral hepatitis (*n* = 5, 17.9%), followed by Wilson Disease (*n* = 4, 14.3%), drug‐induced liver injury (DILI) (*n* = 3, 10.7%), and toxins (*n* = 1, 3.6%); while the cause/trigger for ALF remained unknown in 8 cases (28.5%); without any significant differences between ALF patients with vs. without Cytosorb add‐on treatment. Median SOFA and SAPS II scores within the first 24 h after ICU admission were 11 (IQR: 5–14) and 43 (IQR: 33.3–50.3), respectively. Most patients required vasopressor support (*n* = 23, 82.1% noradrenaline and *n* = 13, 46.4% vasopressin) and invasive mechanical ventilation (IMV; *n* = 24, 85.7%). The primary reason for intubation was HE (*n* = 15, 53.6%), followed by elective intubation for HU‐LTx (*n* = 6, 21.4%) and respiratory failure (*n* = 3, 10.7%). In most patients, CRRT was initiated on the day of ICU admission (IQR: 0–1 days) and continued for 3.5 days (IQR: 2.3–6.8 days). The median time from admission to Cytosorb initiation was 0.5 days (IQR: 0–2.3) with a median duration of 4 (IQR: 2.8–4) days and a median of 2.5 (IQR: 0.3–3.8) adsorber changes. During CRRT, 2 patients in the Cytosorb group received a cycle of plasma exchange, and 3 patients in the control group received a cycle of MARS therapy. Nineteen patients were listed for an HU‐LTx (67.9%), and 13 received an HU‐LTx (46.4%). Median ICU‐LOS was 12 (IQR: 4–19.8) days. Overall ICU survival was 60.7% (*n* = 17), with a 1‐month survival of 53.6% (*n* = 15) and a 6‐month survival of 50% (*n* = 14).

**TABLE 1:** Comparison of clinical and ICU‐specific characteristics.

	All patients	Cytosorb group	Control group	*p* *
Clinical characteristics				
*N* (%)	28 (100)	14 (50)	14 (50)	—
Age, median (IQR)	47.1 (32.5–56.4)	43.2 (27.9–54.6)	52.6 (41.9–58.5)	0.125
Female (%)	23 (82.1)	11 (78.6)	12 (85.7)	0.622
Male (%)	5 (17.9)	3 (21.4)	2 (14.3)	0.622
BMI, median (IQR)	25.5 (22.1–28.9)	25.5 (21.8–28.8)	25.9 (22.2–32.5)	0.768
Aetiology of acute liver failure				
AIH, *n* (%)	7 (25)	3 (21.4)	4 (28.6)	0.663
Viral, *n* (%)	5 (17.9)	3 (21.4)	2 (14.3)	0.622
Wilson, *n* (%)	4 (14.3)	2 (14.3)	2 (14.3)	1
DILI, *n* (%)	3 (10.7)	1 (7.1)	2 (14.3)	0.541
Toxin, *n* (%)	1 (3.6)	0	1 (7.1)	0.309
Unknown, *n* (%)	8 (28.5)	5 (35.7)	3 (21.4)	0.403
ALF onset				
Hyperacute, *n* (%)	10 (35.7)	10 (71.4)	5 (35.7)	0.430
Acute, *n* (%)	15 (53.6)	4 (28.6)	6 (42.9)	0.058
Subacute, *n* (%)	3 (10.7)	0	3 (21.4)	0.067
ICU specific characteristics				
SAPSII**, median (IQR)	43 (33.3–50.3)	38.5 (27.8–44)	45.5 (39.3–60.8)	**0.039**
SOFA**, median (IQR)	11 (5–14)	7 (4.8–14.5)	13 (8.8–14.3)	0.164
ICU‐LOS (days), median (IQR)	12 (4–19.8)	15.5 (4.8–22.5)	7 (4–15)	0.137
Vasopressor therapy, *n* (%)	23 (82.1)	12 (85.7)	11 (78.6)	0.622
Noradrenaline, *n* (%)	23 (82.1)	12 (85.7)	11 (78.6)	0.622
Peak dose NA in μg/kg/min, median (IQR)	0.42 (0.2–0.92)	0.35 (0.21–0.85)	0.52 (0.2–1.19)	0.880
Vasopressin, *n* (%)	13 (46.4)	7 (50)	6 (42.9)	0.705
Peak dose VP in μg/kg/min, median (IQR)	2 (2–3)	2 (2–3)	2 (2–3.3)	0.628
IMV, *n* (%)	24 (85.7)	11 (78.6)	13 (92.9)	0.280
Adm. to IMV (days), median (IQR)	2 (0–3)	2 (2–4)	1 (0–2.5)	**0.038**
Duration of IMV (days), median (IQR)	4.5 (1–8.5)	4 (1–9)	5 (1–10)	0.976
Reason for IMV				
HE, *n* (%)	15 (53.6)	7 (50)	8 (57.1)	0.916
HU‐LTx, *n* (%)	6 (21.4)	3 (21.4)	3 (21.4)	0.813
Respiratory failure, *n* (%)	3 (10.7)	1 (7.1)	2 (14.3)	0.642
ICU survival, *n* (%)	17 (60.7)	10 (71.4)	6 (42.9)	0.127
1‐month survival, *n* (%)	15 (53.6)	10 (71.4)	5 (35.7)	0.058
6‐month survival, *n* (%)	14 (50)	10 (71.4)	4 (28.6)	**0.023**
HU‐LTx, *n* (%)	13 (46.4)	9 (64.3)	4 (28.6)	0.058
HU‐LTx listed (%)	19 (67.9)	13 (92.9)	6 (42.9)	**0.005**
Adm. to HU‐LTx (days), median (IQR)	3 (2–4)	3 (2–5)	2.5 (2–3.8)	0.503
CRRT and Cytosorb				
CRRT				
Adm. to CRRT (days), median (IQR)	0 (0–1)	0 (0–1)	0 (0–1)	0.804
Duration of CRRT (days), median (IQR)	3.5 (2.3–6.8)	3 (2–4)	4 (3–9)	0.164
Citrate accumulation, *n* (%)	14 (50)	7 (50)	7 (50)	1
Bleeding events, *n* (%)	8 (39.3)	4 (28.6)	4 (28.6)	1
Cytosorb				
Adm. to Cytosorb (days), median (IQR)	0.5 (0–2.3)	0.5 (0–2.3)	—	—
Duration of Cytosorb (days), median (IQR)	4 (2.8–4)	4 (2.8–4)	—	—
Cytosorb changes, median (IQR)	2.5 (0.3–3.8)	2.5 (0.3–3.8)	—	—
MARS therapy during ICU stay, *n* (%)	3 (10.7)	0	3 (21.4)	0.067
HVPE during ICU stay, *n* (%)	2 (7.1)	2 (14.3)	0	0.142

*Note:* Bold values highlight statistically significant results.

Abbreviations: AIH, autoimmune hepatitis; ALF, acute liver failure; BMI, body mass index; CRRT, continuous renal replacement therapy; DILI, drug‐induced liver injury; HE, hepatic encephalopathy; HU‐LTx, high urgency liver transplantation; HVPE, high‐volume plasma exchange; IMV, invasive mechanical ventilation; IQR, interquartile range; MARS, molecular adsorbent recirculating system; SAPSII, simplified acute physiology score II; SOFA, Sequential Organ Failure Assessment.

* *p*‐values were defined as statistically significant when *p* < 0.05, without consideration of multiple comparisons.

** SAPSII and SOFA scores were calculated within the first 24 h after admission.

**TABLE 2:** Patients' characteristics.

Group	Pt. *n*	Age	M/F	ALF aetiology	ALF type	SOFA Score^a^	SAPS II Score^a^	ICU‐LOS	IMV^b^	Reason for IMV	VP^b^	CA	HVPE	MARS	HU‐LTx	ICU Survival	1‐month survival	6‐month survival	Reason for death
CRRT + Cytosorb	1	35	F	UK	Acute	4	22	68	Yes	HE	Yes	Yes	No	No	Yes	Yes	Yes	Yes	—
	2	34	F	UK	Acute	4	28	14	No	—	No	No	No	No	No	Yes	Yes	Yes	—
	3	47	F	Viral	Acute	9	33	4	Yes	HE	Yes	Yes	No	No	No	No	No	No	MOF
	4	44	M	Viral	Hyperacute	5	36	22	Yes	HU‐LTx	Yes	No	No	No	Yes	Yes	Yes	Yes	—
	5	22	M	M. Wilson	Acute	14	27	17	Yes	HU‐LTx	Yes	No	No	No	Yes	Yes	Yes	Yes	—
	6	30	F	Viral	Hyperacute	16	41	27	Yes	HE	Yes	Yes	Yes	No	Yes	Yes	Yes	Yes	—
	7	19	F	AIH	Acute	5	20	10	Yes	HE	Yes	Yes	No	No	Yes	No	No	No	MOF
	9	44	F	UK	Acute	5	42	24	Yes	HE	Yes	No	No	No	Yes	Yes	Yes	Yes	—
	18	43	F	UK	Acute	18	48	14	Yes	HU‐LTx	Yes	Yes	No	No	Yes	Yes	Yes	Yes	—
	10	22	M	M. Wilson	Hyperacute	14	36	5	Yes	RF	Yes	No	No	No	No	No	No	No	MOF
	11	57	F	AIH	Acute	5	43	20	Yes	HE	Yes	Yes	No	No	Yes	Yes	Yes	Yes	—
	12	54	F	DILI	Acute	2	52	4	No	—	No	No	No	No	No	Yes	Yes	Yes	—
	13	60	F	AIH	Hyperacute	16	43	4	No	—	Yes	Yes	No	No	No	No	No	No	MOF
	14	61	F	UK	Acute	10	47	19	Yes	HE	Yes	No	Yes	No	Yes	Yes	Yes	Yes	—
CRRT	15	52	F	M. Wilson	Acute	9	41	21	Yes	HU‐LTx	Yes	No	No	No	Yes	Yes	Yes	Yes	—
	16	32	F	AIH	Hyperacute	8	34	9	Yes	HE	No	No	No	No	No	Yes	Yes	Yes	—
	17	54	F	AIH	Acute	9	32	81	Yes	HE	Yes	Yes	No	No	Yes	No	Yes	No	ICB
	18	59	F	UK	Hyperacute	15	47	4	Yes	HU‐LTx	Yes	No	No	No	No	Yes	Yes	Yes	—
	19	32	M	M. Wilson	Hyperacute	14	43	15	Yes	HU‐LTx	Yes	Yes	No	Yes	Yes	Yes	Yes	Yes	—
	20	27	F	DILI	Acute	7	21	8	No	—	No	Yes	No	Yes	No	Yes	No	No	CF
	21	55	F	Toxin	Hyperacute	12	44	2	Yes	HE	Yes	Yes	No	No	No	No	No	No	MOF
	22	70	F	Viral	Hyperacute	17	69	6	Yes	HE	Yes	Yes	No	Yes	No	No	No	No	MOF
	23	59	F	DILI	Acute	14	79	15	Yes	HE	Yes	No	No	No	No	No	No	No	MOF
	24	53	F	UK	Subacute	13	51	4	Yes	RF	Yes	No	No	No	No	No	No	No	MOF
	25	45	F	Viral	Acute	14	58	1	Yes	HE	Yes	Yes	No	No	No	No	No	No	MOF
	26	47	M	UK	Hyperacute	13	54	5	Yes	HE	No	No	No	No	No	No	No	No	MOF
	27	53	F	AIH	Subacute	21	70	15	Yes	RF	Yes	No	No	No	No	Yes	No	No	MOF
	28	59	F	AIH	Subacute	5	43	4	Yes	HE	Yes	Yes	No	No	Yes	No	No	No	HS

Abbreviations: AIH, autoimmune hepatitis; ALF, acute liver failure; CA, citrate accumulation; CF, cardiovascular failure; CRRT, continuous renal replacement therapy; DILI, drug‐induced liver injury; HE, hepatic encephalopathy; HS, hemorrhagic shock; HU‐LTx, high urgency liver transplantation; HVPE, high‐volume plasma exchange; ICB, intracranial bleeding; IMV, invasive mechanical ventilation; MARS, molecular adsorbent recirculating system; MOF, multi‐organ failure; RF, respiratory failure; SAPS II, Simplified Acute Physiology Score II; SOFA, Sequential Organ Failure Assessment; UK, unknown; VP, vasopressor.

^a^ SAPSII and SOFA scores were calculated within the first 24 h after ICU admission.

^b^ IMV and Vasopressor therapy during ICU stay.

### 3.2 CRRT and Cytosorb vs. CRRT Only

Differences in baseline characteristics between patients receiving CRRT and Cytosorb and patients with CRRT only are shown in Table 1. Patients in the Cytosorb group had lower SOFA (7 vs. 13, *p* = 0.164) and SAPS II (38.5 vs. 45.5, *p* = 0.039) scores. The median time to intubation was prolonged in the Cytosorb group (2 vs. 1 day, *p* = 0.038). There was no significant difference in ICU‐specific therapies between both groups. There was a trend toward a longer ICU‐LOS in the Cytosorb group (15.5 vs. 7 days, *p* = 0.137). Patients receiving Cytosorb therapy were significantly more often listed for HU‐LTx (92.9 vs. 42.9%, *p* = 0.005) and, therefore, more frequently transplanted (64.3 vs. 28.6% *p* = 0.058), resulting in a significantly higher long‐term survival rate in the Cytosorb group, with a 6‐month survival rate of 71.4% compared to 28.6% in the control group (*p* = 0.023). However, there was no significant impact on ICU (71.4 vs. 42.9%, *p* = 0.127) and 1‐month (71.4 vs. 35.7%, *p* = 0.058) survival (see also Figure S4).

### 3.3 Trajectory of Laboratory Parameters During Cytosorb Therapy

**FIGURE 2:**
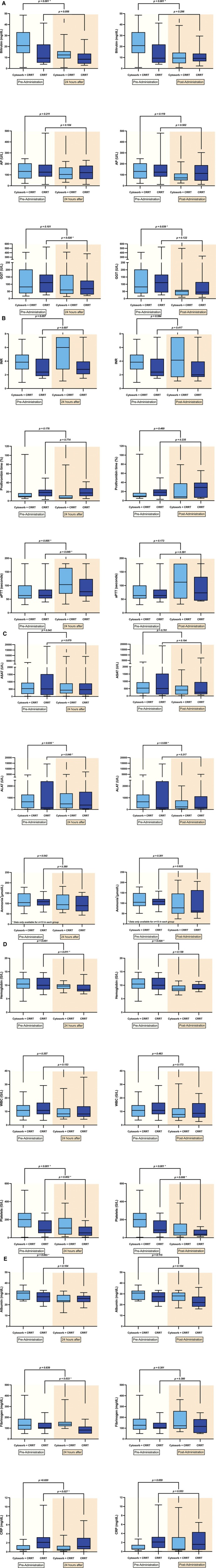
(A–E) Course of laboratory parameters during CRRT and Cytosorb treatment. Median values of laboratory parameters were given as boxplots with whiskers in the CRRT + Cytosorb group in a light blue box and the CRRT group in a dark blue box. Laboratory values are given pre‐treatment, after 24 h, and post‐treatment. Wilcoxon signed rank test was given for median changes in both groups from pre‐treatment to after 24 h or pre‐treatment to post‐treatment. P‐values with *p* < 0.05 were determined as significant and marked with a star. Laboratory values are shown for bilirubin, AP and gamma‐GT (A), INR, Prothrombin time and aPTT (B), ASAT, ALAT, and Ammonia (only for *n* = 13 in each group) (C), Hemoglobin, WBC and Platelets (D) and Albumin, Fibrinogen, and CRP (E). Laboratory values were available for all patients (*n* = 28) except for ammonia (*n* = 26). ALAT, alanine aminotransferase; aP, alkaline phosphatase; aPTT, activated partial thromboplastin time; ASAT, aspartate aminotransferase; CRP, c‐reactive protein; CRRT, continuous renal replacement therapy; GGT, gamma‐glutamyl transferase; INR, international normalised ratio; WBC, white blood count.

Changes in laboratory parameters upon Cytosorb treatment are depicted in Figure 2A–E and Table S1. A significant reduction of bilirubin levels was observed after 24 h of Cytosorb therapy (median: 20.85 to 12.32 mg/dL, *p* < 0.001) and after completing Cytosorb treatment (median: 20.85 to 9.46 mg/dL, *p* < 0.001). We also found a significant decrease in GGT levels post‐treatment (median: 81.5 to 51 U/L, *p* = 0.039) and ALAT levels after 24 h (median: 658 to 479.5 U/L, *p* = 0.035) and after treatment (median: 658 to 228.5 U/L, *p* = 0.020). Median ammonia levels, only available in 13 patients in each group, were decreasing after 24 h (median: 104.4 to 94.3 μmol/L, *p* = 0.542) and after treatment (median: 104.4 to 77.6 μmol/L, *p* = 0.301), but with no statistical significance.

Moreover, median WBCs were lower after 24 h and post‐treatment. However, with no statistical significance. CRP levels were consistently low throughout the ICU stay, and Cytosorb therapy had no impact on its course. Data on IL‐6 and PCT measurements were available for only 7 and 6 patients, respectively (Table S1). A change in the blood coagulation parameters aPTT, prothrombin time, and INR was detected during Cytosorb treatment. A significant decline in platelet counts was observed during (median: 201.5 to 107 G/L, *p* < 0.001) and after Cytosorb treatment (median: 201.5 to 54 G/L, *p* < 0.001). During Cytosorb treatment we noted stable lactate levels and noradrenaline requirements (Figure S3).

Laboratory changes were also assessed in the control group to determine whether they were attributed to additional Cytosorb treatment or conventional CRRT alone. While a decrease in bilirubin levels was only found in the Cytosorb group, decreasing levels of ammonia, platelets, creatinine, and BUN were observed in both groups. Laboratory changes in various parameters are depicted in Figure 2A–E, Figures S2 and S3 and Table S1.

### 3.4 Feasibility, Safety, and Complications of Cytosorb Therapy in ALF Patients

**FIGURE 3:**
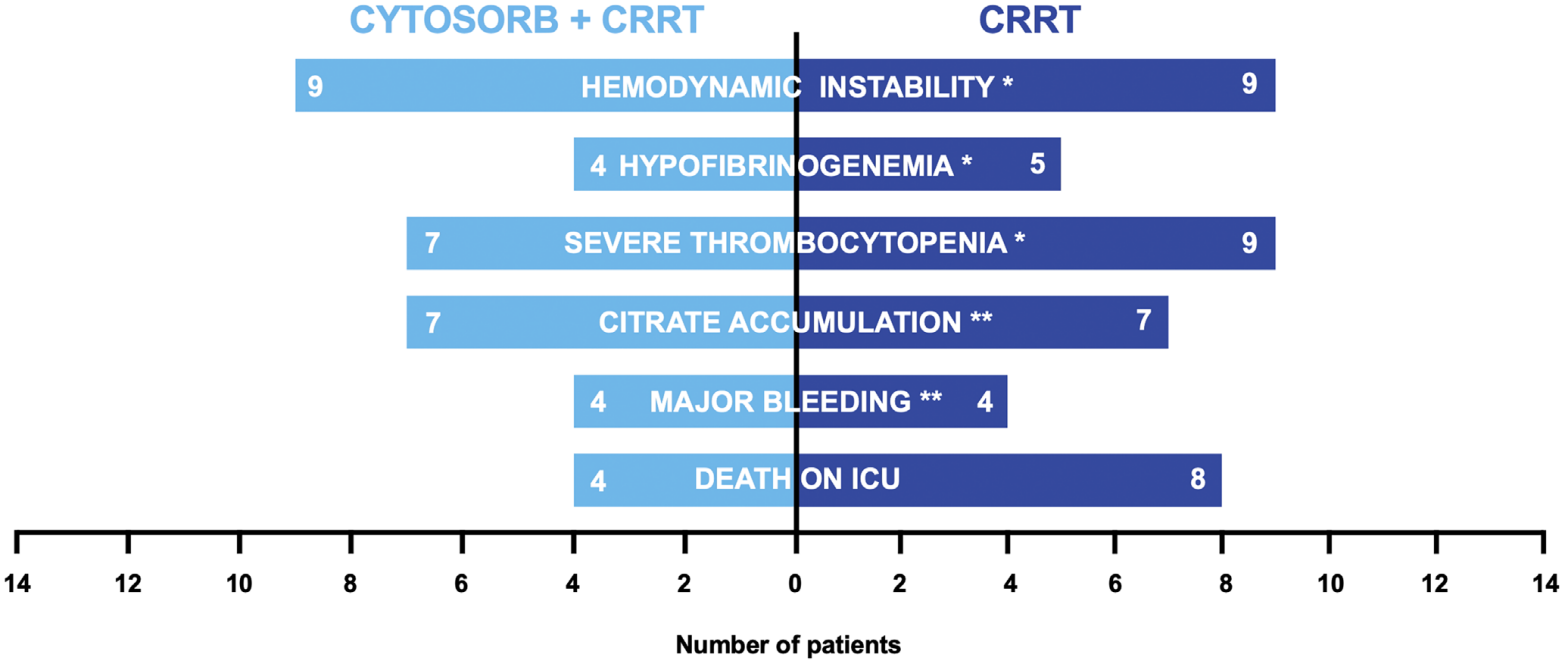
Adverse events during/after Cytosorb and CRRT treatment. Absolute numbers of patients were given in bars with light blue in the Cytosorb + CRRT group and dark blue in the CRRT group. Hemodynamic instability was defined as vasopressor support or mean blood pressure < 65 mmHg, observed hypofibrinogenemia as fibrinogen levels < 100 mg/dL, severe thrombocytopenia as platelets below < 50 G/L, citrate accumulation as signs of citrate accumulation (i.e., total calcium to ionised calcium ratio > 2.5) and major bleeding events as bleedings fatal bleedings, bleeding with a decrease in haemoglobin level of ≥ 2 g/dL or bleeding leading to transfusion of ≥ 2 units of packed red blood cells. *Hemodynamic instability, hypofibrinogenemia, and severe thrombocytopenia were investigated at the time of Cytosorb/CRRT discontinuation. **Citrate accumulation and major bleeding events were investigated during Cytosorb/CRRT treatment.

Individual patients' courses and information on observed adverse events potentially associated with CRRT and Cytosorb or CRRT alone are depicted in Figures 1 and 3. Hemodynamic instability was observed in both groups (both groups: *n* = 9, 64.3%) with no statistically significant difference. As potential markers for clotting and coagulation, we observed hypofibrinogenemia (Cytosorb: *n* = 4, 28.6% vs. control group: *n* = 5, 35.7%) and severe thrombocytopenia (Cytosorb: *n* = 7, 50% vs. control group: *n* = 9, 64.3%). While major bleedings occurred in 8 patients (*n* = 5 gastrointestinal bleeding, *n* = 1 tracheal bleeding, *n* = 1 perioperative bleeding, *n* = 1 intraabdominal bleeding), there was no statistically significant difference between the two groups. Interestingly, citrate accumulation occurred in 50% of the study population, relatively early during CRRT, with no difference between the two groups. No other complications, such as allergic reactions, were observed in our ALF patient cohort that could be attributed to treatment with the Cytosorb adsorber.

## 4 Discussion

In this retrospective study, we aimed to evaluate the safety and feasibility of Cytosorb treatment in ALF patients. In our cohort, 14 of 28 patients received Cytosorb adsorbers during their ICU stay. Among those patients, we observed a significant decrease in bilirubin, GGT, and ALAT post‐treatment, which was evident as early as after 24 h. Platelet counts were significantly decreased after 24 h and following treatment—but decreasing platelet counts were also noted in ALF patients with CRRT only; that is, without additional Cytosorb treatment. Importantly, the requirement for noradrenaline support and lactate levels remained stable during Cytosorb treatment. Thus, in our hands we find the add‐on use of Cytosorb adsorber on top of CRRT in patients with ALF to be a feasible and easy‐to‐use blood purification tool without any major safety concerns—as compared to CRRT alone.

Over the last decades, several artificial and bioartificial liver‐assist devices have been studied with an attempt to support liver function in patients with liver failure [6, 7, 8, 9, 10]. However, no robust evidence supporting a clinically relevant benefit was reported [3]. HVPE is the only adjunctive extracorporeal therapy recommended in ALF patients by the current EASL guidelines [1]. This “grade 2 recommendation” was supported primarily by evidence from a single clinical trial demonstrating improved transplant‐free survival, particularly among patients ineligible for HU‐LTx [12]. In a recent real‐world ALF cohort, plasma exchange showed improvements in hemodynamics but did not improve survival [24]. Consequently, the effects of plasma exchange are still debatable, and more data are needed. While some devices, such as MARS, Prometheus, and OPAL, led to an improvement in laboratory parameters, they failed to improve survival or to support bridging to transplantation or recovery [1, 6, 7, 8, 9, 10]. Evidence comparing all available systems remains limited. Nevertheless, recent data indicate that Cytosorb demonstrates superior efficacy in bilirubin removal and enhanced clearance of albumin‐bound toxins and cytokines (e.g., IL‐6 and TNF‐α) compared to MARS [25]. Unlike MARS, which primarily removes direct bilirubin without significant impact on its indirect form, Cytosorb facilitates near‐complete removal of both bilirubin subfractions [26]. Furthermore, the high cost of MARS/Prometheus/OPAL and the requirement of well‐trained medical staff limit the use of these devices to highly specialized (transplantation) centres [3, 6, 7, 11]. In contrast, Cytosorb is an easily applicable adsorber that can be added to conventional CRRT, not requiring significant expertise [13, 27, 28, 29, 30]. It was first introduced for the modulation of hyperinflammation in sepsis [31]. The value of adjunctive Cytosorb treatment in sepsis is still heavily debated due to the lack of large prospective trials. The interpretation of available data is also limited by different application times and change intervals of the Cytosorb cartridge [32, 33].

The rationale for using Cytosorb in liver failure is based on its ability to remove circulating toxins (e.g., bilirubin, bile acids) and proinflammatory cytokines that accumulate due to impaired hepatic clearance and liver damage, contributing to multiorgan failure and poor outcomes [4, 5, 30]. Thereby, Cytosorb may help to mitigate systemic inflammation—a key driver of severe liver‐related complications—and organ dysfunction [34]. By reducing the burden of circulating toxins in ALF patients, Cytosorb might serve as a potential bridge to liver transplantation or recovery. A significant impact on markers for systemic inflammation (i.e., IL‐6 and PCT) has already been observed among ACLF patients [13]. In this study, we only had IL‐6 levels available in a subgroup of patients, limiting our analysis and conclusions. However, among those patients with elevated IL‐6 before Cytosorb treatment, levels decreased after Cytosorb treatment. Notably, Cytosorb also significantly reduced serum levels of GGT after Cytosorb treatment, which serves as a non‐specific, early, and highly sensitive marker of oxidative stress in liver failure [35]. The potential decrease in cytokine levels might have been involved in the observed hemodynamic stabilization of ALF patients treated by Cytosorb—as suggested by stable noradrenaline requirements and lactate levels during treatment.

Furthermore, bilirubin was significantly reduced by the Cytosorb adsorber in our ALF patients. An effect on bilirubin levels, although less pronounced, was previously also reported in ALF patients receiving MARS [13, 15, 25, 27, 28, 29, 30]. Besides jaundice and pruritus, hyperbilirubinemia is discussed to have neurotoxic effects in case of a disrupted blood–brain barrier [36, 37]. Thus, the dampening of severe hyperbilirubinemia using Cytosorb could also be beneficial in cases of ALF by supporting the prevention of brain edema and cholestasis‐related complications in addition to facilitating hemodynamic stability before HU‐LTx [38]. A corresponding neuroprotective effect might be underlined by significantly delayed intubation among patients receiving Cytosorb therapy (2 vs. 1 day). The impact of Cytosorb on ammonia levels is still debated [16, 25, 39]. However, our data suggest that the decrease in ammonia levels is mainly achieved by CRRT alone [40], as we did not find any significant differences between our subgroups with vs. without add‐on Cytosorb therapy.

We found a significant decrease in platelets during and after Cytosorb therapy– which was also seen in ALF patients only receiving CRRT. Thrombocytopenia observed during Cytosorb treatment may result from several mechanisms. The adsorber's polymer beads can induce platelet activation and adhesion, leading to mechanical removal or consumption of platelets within the cartridge. In addition, shear stress generated by blood flow through the adsorber and tubing may cause mechanical platelet damage and enhance platelet aggregation or fragmentation, further contributing to platelet depletion. Activation of the complement and contact systems, as well as adsorption of platelet‐activating mediators on the Cytosorb surface, may exacerbate this effect [41]. These mechanisms are comparable to those described in other extracorporeal modalities such as MARS, CRRT or ECMO [6, 42, 43, 44, 45, 46, 47]. Our findings of decreased platelet levels under Cytosorb treatment are in line with previously published publications [13, 16]. These changes were accompanied by fulminant coagulation failure in both subgroups, findings which are best explained by the disease progression of hepatic failure itself and the critical illness in ALF, further contributing to thrombopenia [48, 49]. Still, the effect of declining platelets requires further investigation, as it might resemble a relevant marker for survival in ALF patients [3, 48, 49]. While ALF patients are generally affected by low fibrinogen levels due to severe liver impairment, we detected no significant decrease in fibrinogen levels during Cytosorb treatment [50].

In our cohort, we did not detect a higher rate of complications associated with Cytosorb than in patients only receiving conventional dialysis. Moreover, we did not detect technical issues, installation problems, or clotting with Cytosorb. Deranged coagulation and prothrombotic risk during extracorporeal therapy ± Cytosorb may also be attributed to ALF disease progression and multiorgan dysfunction. Bleeding events during CRRT occurred relatively frequently (39.3%) compared to a recent registry study, which reported an overall bleeding rate of only 11%—still considered infrequent relative to preexisting literature [3, 51].

The Cytosorb group had a significantly increased 6‐month survival compared to the control group. However, these findings need to be interpreted with caution, as patients receiving Cytosorb treatment had lower disease severity scores (as reflected by SAPS II) and, more importantly, showed a higher rate of HU‐LTx listing, and consequently higher rates of actual HU‐LTx. For now, it is unclear to what extent Cytosorb treatment contributed to clinical stabilisation and readiness for potential HU‐LTx.

Our study has some major limitations: First, this study's retrospective design inherently limits the ability to establish causal relationships. Furthermore, potential selection bias and incomplete data collection may have influenced the results and should be considered when interpreting the findings. Second, due to the single‐centre study, we can only report a small cohort of 14 patients receiving Cytosorb treatment. However, to our knowledge, this is the first study to specifically examine the effects of hemoadsorption with Cytosorb in patients with ALF. Third, due to the retrospective study design, missing data on IL‐6 and PCT resulted in inconclusive findings. Fourth, the effect of Cytosorb was only conducted in conjunction with standard intensive care treatment and CRRT (± MARS/HVPE in a small subset of patients), which may bias the impact of Cytosorb treatment. Fifth, due to the small sample size, adequate matching to the control group was not feasible. Importantly, patients in the Cytosorb group had a significantly higher 6‐month survival rate compared to the control group. However, this may be primarily attributable to higher listing and transplantation rates in the Cytosorb group. Comparability between groups is therefore limited. Future multicentre prospective trials are required to more comprehensively compare the efficacy of CRRT combined with Cytosorb therapy versus CRRT alone in patients with ALF. Nevertheless, we provide a comprehensive overview of our single‐centre experience with Cytosorb adsorber therapy in ALF patients.

## 5 Conclusion

In this single‐centre cohort of ALF patients, CRRT combined with Cytosorb therapy was associated with significant reductions in bilirubin levels, liver enzymes, and platelet counts, as well as trends toward clinical stabilisation. The data of this study highlight the safety and feasibility of Cytosorb therapy in ALF patients as it was generally well tolerated, with no relevant signals related to safety issues and an easy‐to‐use add‐on to CRRT. However, efficacy signals on the clinical outcome in patients with ALF still remain preliminary and confounded, due to the small sample size, retrospective study design and patient selection. Larger prospective studies are warranted in ALF patients to assess the actual clinical value of Cytosorb therapy as a potential extracorporeal assist device in ALF patients.

## Author Contributions

Concept of the study: H.P., Z.C., S‐.G.M. Data collection: H.P., F.S., R.R., S‐.G.M. Statistical analysis: H.P., S‐.G.M. Drafting of the manuscript: H.P., S‐.G.M. Revision for important intellectual content as well as approval of the final manuscript: All authors.

## Ethics Statement

The study was conducted according to the guidelines of the Declaration of Helsinki. It was approved by the local Ethics Committee of the Medical University of Vienna with the Ethics committee number: 1229/2024. Moreover, the responsible Ethics committee waived the informed consent requirement, due to the retrospective setting of the study design.

## Conflicts of Interest

R.T. received grant support from AbbVie, Boehringer Ingelheim, Gilead, Intercept/Advanz Pharma, MSD, Myr Pharmaceuticals, Philips Healthcare, Pliant, Siemens and W. L. Gore & Associates; speaking/writing honoraria from AbbVie, Echosens, Gilead, GSK, Intercept/Advanz Pharma, Pfizer, Roche, MSD, Siemens, W. L. Gore & Associates; consulting/advisory board fees from AbbVie, Astra Zeneca, Bayer, Boehringer Ingelheim, Gilead, Intercept/Advanz Pharma, MSD, Resolution Therapeutics, Siemens; and travel support from AbbVie, Boehringer Ingelheim, Dr. Falk Pharma, Gilead, and Roche. T.M. received grant support from Albireo, Alnylam, Cymabay, Falk, Genentech, Gilead, Intercept, MSD, Takeda and UltraGenyx; honoraria for consulting from AbbVie, Albireo, Agomab, Alfasigma, Boehringer Ingelheim, BiomX, Chemomab, Dexoligo Therapeutics, Falk, Genfit, Gilead, GSK, Hightide, Intercept, Ipsen, Janssen, MSD, Mirum, Novartis, Phenex, Pliant, ProQR Therapeutics, Regulus, Siemens and Shire; speaker fees from Albireo, Boehringer Ingelheim, Bristol‐Myers Squibb, Falk, Gilead, Ipsen, Intercept, Madrigal, MSD and Mirum; as well as travel support from AbbVie, Falk, Gilead, Janssen, Intercept and Ipsen. He is also a co‐inventor of patents on the medical use of 24‐norursodeoxycholic acid (service inventions as a university employee) filed by the Medical University of Graz. S‐.G.M. received speaker fees from Cytosorbents.

## Supporting information


Data S1.

## Data Availability

Data are available from the corresponding author upon reasonable request.
