# Supplementary material for: Feasibility and Safety of Cytosorb Application in Patients With Acute Liver Failure
Source: Liver Int. 2025 Nov 14;45(12):e70420. doi: 10.1111/liv.70420 (PMC12617026; doi:10.1111/liv.70420)
Supplement: Supplementary file 1 — Data S1. [file LIV-45-0-s001.docx]

**Supplementary Material**

**Feasibility and Safety of Cytosorb application in patients with acute liver failure.**

Haselwanter Patrick^1^, Fairfield Seanna^1^, Reinhold Riva^1^, Riedl-Wewalka Marlene^1^, Schmid Monika^1^, Balcar Lorenz^1^, Stättermayer Albert Friedrich^1^, Reiberger Thomas^1,2,3^, Trauner Michael^1,2^, Zauner Christian^1^, Schneeweiss-Gleixner Mathias^1^

^1^ Department of Medicine III, Division of Gastroenterology and Hepatology, Intensive Care Unit 13H1, Medical University of Vienna, Austria

^2^ Clinical Research Group MOTION, Medical University of Vienna, Vienna, Austria

^3^ Christian-Doppler Laboratory for Portal Hypertension and Liver Fibrosis, Medical University of Vienna, Vienna, Austria

**Supplemental Table S1:** Trajectory of laboratory & hemodynamic parameters in Cytosorb and Control group.

| **Median laboratory parameters (IQR)** | **Cytosorb group** | **delta Δ** | ***p-value*** | **Control group** | **delta Δ** | ***p-value*** |
| --- | --- | --- | --- | --- | --- | --- |
| **Bilirubin n (%)** | 14 (100) |  |  | 14 (100) |  |  |
| *Pre administration in mg/dL* | 20.85 (14.10-33.07) |  |  | 9.37 (5.28-21.88) |  |  |
| *24 hours after in mg/dL* | 12.32 (9.18-15.75) | *-8.53* | **0.0001** | 8.45 (4.41-13.54) | *-0.92* | 0.0559 |
| *Post administration in mg/dL* | 9.46 (4.8-14.44) | *-11.39* | **0.0001** | 9.24 (6.55-13.59) | *-0.13* | 0.2958 |
| **AP n (%)** | 14 (100) |  |  | 14 (100) |  |  |
| *Pre administration in U/L* | 131 (67-204.8) |  |  | 124 (83.75-191.5) |  |  |
| *24 hours after in U/L* | 103.5 (61.5-167.5) | *-27.5* | 0.2106 | 120 (61.50-183.8) | *-4* | 0.1040 |
| *Post administration in U/L* | 72 (50-111.5) | *-59* | 0.1189 | 113.0 (46.75-188.3) | *-11* | 0.5016 |
| **GGT n (%)** | 14 (100) |  |  | 14 (100) |  |  |
| *Pre administration in U/L* | 81.5 (37-202.5) |  |  | 111 (43.25-166.5) |  |  |
| *24 hours after in U/L* | 60.50 (33.75-164.5) | *-21* | 0.1005 | 69 (29.25-125) | *-42* | **0.0203** |
| *Post administration in U/L* | 51 (23.5-60.25) | *-30.5* | **0.0393** | 45.5 (29.25-115.3) | *-65.5* | 0.1223 |
| **INR n (%)** | 14 (100) |  |  | 14 (100) |  |  |
| *Pre administration* | 3.85 (2.8-4.95) |  |  | 2.4 (1.83-4.35) |  |  |
| *24 hours after* | 6 (2.8-7.5) | *2.15* | 0.0574 | 2.8 (2.1-3.95) | *0.4* | 0.6973 |
| *Post administration* | 4.15 (1.68-7.5) | *0.3* | 0.5936 | 2 (1.7-3.95) | *-0.4* | 0.4169 |
| **Prothrombin time n (%)** | 14 (100) |  |  | 14 (100) |  |  |
| *Pre administration in %* | 10 (8-17) |  |  | 18.5 (10.25-24.5) |  |  |
| *24 hours after in %* | 6.5 (5-12) | *-3.5* | 0.1758 | 17.5 (10.5-27.75) | *-1* | 0.7744 |
| *Post administration in %* | 8.5 (5-38) | *-1.5* | 0.4697 | 29 (6.5-39.5) | *10.5* | 0.2355 |
| **aPTT n (%)** | 14 (100) |  |  | 14 (100) |  |  |
| *Pre administration in s* | 63.4 (52.75-100.5) |  |  | 63.5 (53.20-85.05) |  |  |
| *24 hours after in s* | 103.5 (70.13-164.5) | *40.1* | **0.0052** | 77.4 (60.63-124.1) | *13.9* | 0.0494 |
| *Post administration in s* | 112.4 (52.7-180) | *49* | 0.1726 | 73.3 (45.05-131.9) | *9.8* | 0.3910 |
| **ASAT n (%)** | 14 (100) |  |  | 14 (100) |  |  |
| *Pre administration in U/L* | 537 (175-938) |  |  | 518.5 (108-1527) |  |  |
| *24 hours after in U/L* | 431 (213.3-895) | *-106* | 0.5416 | 470 (126.5-892) | *-48.5* | **0.0785** |
| *Post administration in U/L* | 407 (107.5-704.8) | *-130* | 0.7609 | 256 (125.8-952) | *-262.5* | 0.1937 |
| **ALAT n (%)** | 14 (100) |  |  | 14 (100) |  |  |
| *Pre administration in U/L* | 658 (117.3-1271) |  |  | 168.5 (34.5-2407) |  |  |
| *24 hours after in U/L* | 479.5 (106.3-1380) | *-178.5* | **0.0353** | 378.5 (41.5-1508) | *210* | **0.0494** |
| *Post administration in U/L* | 228.5 (61.5-772.5) | *-429.5* | **0.0203** | 180 (58.25-1125) | *11.5* | 0.2166 |
| **Ammonia n (%)** | 13 (92.8) |  |  | 13 (92.8) |  |  |
| *Pre administration in µmol/L* | 104.4 (86.35-148.7) |  |  | 108.9 (90.75-120.9) |  |  |
| *24 hours after in µmol/L* | 94.3 (70.3-143.1) | *-10.1* | 0.5417 | 89.5 (62.65-136.5) | *-19.4* | 0.3804 |
| *Post administration in µmol/L* | 77.6 (49.8-146.7) | *-26.8* | 0.3013 | 62.3 (54.9-163) | *-46.6* | 0.6221 |
| **Hemoglobin n (%)** | 14 (100) |  |  | 14 (100) |  |  |
| *Pre administration in g/dL* | 10.45 (8.78-12.5) |  |  | 10 (8.45-12.73) |  |  |
| *24 hours after in g/dL* | 9.7 (8.7-10.33) | *-0.75* | 0.0508 | 8.45 (7.75-10.2) | *-1.55* | **0.0111** |
| *Post administration in g/dL* | 8.6 (7.88-9.9) | *-1.85* | **0.0200** | 8.75 (8.48-10.43) | *-1.25* | 0.1396 |
| **WBC n (%)** | 14 (100) |  |  | 14 (100) |  |  |
| *Pre administration in G/L* | 10.81 (6.278-14.67) |  |  | 10.84 (8.01-16.52) |  |  |
| *24 hours after in G/L* | 8.23 (5.878-12.35) | *-2.58* | 0.3575 | 8.16 (6.098-13.85) | *-2.68* | 0.1353 |
| *Post administration in G/L* | 7.83 (5.52-12.35) | *-2.98* | 0.4631 | 8.66 (5.528-16.14) | *-2.18* | 0.1726 |
| **Platelets n (%)** | 14 (100) |  |  | 14 (100) |  |  |
| *Pre administration in G/L* | 201.5 (115-273.3) |  |  | 84.5 (51-191.3) |  |  |
| *24 hours after in G/L* | 107 (35.25-217.8) | *-94.5* | **0.0009** | 54 (22-122.3) | *-30.5* | **0.0023** |
| *Post administration in G/L* | 54 (26.25-155) | *-147.5* | **0.0002** | 38 (23-88.75) | *-46.5* | **0.0084** |
| **Albumin n (%)** | 14 (100) |  |  | 14 (100) |  |  |
| *Pre administration in mg/dL* | 30.75 (24.53-32.95) |  |  | 27.3 (22.58-31.45) |  |  |
| *24 hours after in mg/dL* | 23.7 (22.45-29) | *-7.05* | **0.0031** | 25.8 (22.5-28.23) | *-1.5* | 0.1040 |
| *Post administration in mg/dL* | 28.1 (23.6-31.18) | *-2.65* | 0.1145 | 22.05 (18.2-27.48) | *-5.25* | 0.1040 |
| **Fibrinogen n (%)** | 14 (100) |  |  | 14 (100) |  |  |
| *Pre administration in mg/dL* | 127 (87-186.3) |  |  | 104 (95.5-149.5) |  |  |
| *24 hours after in mg/dL* | 136 (118.5-157.8) | *9* | 0.8394 | 82.5 (50-114.3) | *-21.5* | **0.0327** |
| *Post administration in mg/dL* | 122.5 (94.75-258.5) | *-4.5* | 0.5830 | 116.5 (56-183) | *12.5* | 0.8207 |
| **CRP n (%)** | 14 (100) |  |  | 14 (100) |  |  |
| *Pre administration in mg/dL* | 0.56 (0.45-1.47) |  |  | 2.11 (0.8-3.24) |  |  |
| *24 hours after in mg/dL* | 0.56 (0.31-1.34) | *0* | 0.8196 | 1.18 (0.6-3.09) | *-0.93* | **0.0269** |
| *Post administration in mg/dL* | 0.56 (0.31-3.18) | *0* | 0.6586 | 1.59 (0.39-4.32) | *-0.52* | 0.5532 |
| **IL-6 n (%)** | 7 (50) |  |  | - | *-* | - |
| *Pre administration in pg/dL* | 122 (11.81-230) |  |  |  |  |  |
| *24 hours after in pg/dL* | 294.1 (110.9-959.3) | *172.1* | 0.6875 |  |  |  |
| *Post administration in pg/dL* | 158.2 (14.15-474) | *36.2* | 0.9375 |  |  |  |
| **Procalcitonin n (%)** | 6 (42.9) |  |  | - | *-* | - |
| *Pre administration in ng/mL* | 0.39 (0.12 - 4.01) |  |  |  |  |  |
| *24 hours after in ng/mL* | 0.74 (0.18-14.85) | *0.35* | 0.8438 |  |  |  |
| *Post administration in ng/mL* | 8.18 (0.26-31.2) | *7.79* | 0.1562 |  |  |  |
| **Creatinine n (%)** | 14 (100) |  |  | 14 (100) |  |  |
| *Pre administration in mg/dL* | 1.25 (0.66-1.56) |  |  | 1.51 (0.7-2.71) |  |  |
| *24 hours after in mg/dL* | 0.93 (0.58-1.24) | *-0.32* | **0.0101** | 1.49 (0.64-1.9) | *-0.02* | **0.0175** |
| *Post administration in mg/dL* | 0.98 (0.52-1.46) | *-0.27* | 0.2223 | 1.07 (0.63-1.54) | *-0.44* | **0.0203** |
| **BUN n (%)** | 14 (100) |  |  | 14 (100) |  |  |
| *Pre administration in mg/dL* | 17.65 (4.63-39.63) |  |  | 24.85 (9.88-37.83) |  |  |
| *24 hours after in mg/dL* | 10.85 (5.53-20.7) | *-6.8* | **0.0016** | 19.3 (6.6-33.3) | *-5.55* | **0.0166** |
| *Post administration in mg/dL* | 11.2 (5.2-29.7) | *-6.45* | 0.1531 | 13.7 (2.5-25.9) | *-11.15* | **0.0193** |
| **Hemodynamic parameters (IQR)** | **Cytosorb group** | **delta Δ** | ***p-value*** | **Control group** | **delta Δ** | ***p-value*** |
| **Lactate n (%)** | 14 (100) |  |  | 14 (100) |  |  |
| *Pre administration in mmol/L* | 3.85 (2.4-4.759 |  |  | 3.55 (1.95-9.75) |  |  |
| *24 hours after in mmol/L* | 2 (1.38-4.95) | *-1.85* | 0.7035 | 2.5 (1.25-6.93) | *-1.05* | 0.1709 |
| *Post administration in mmol/L* | 2.35 (1.43-4.63) | *-1.5* | 0.5312 | 5.15 (1.38-10.6) | *1.6* | 0.7764 |
| **Noradrenaline n (%)** | 8 (57.1) / 10 (71.4) |  |  | 8 (57.1) / 10 (71.4) |  |  |
| *Pre administration in µg/kg/min* | 0.044 (0-0.153) |  |  | 0.1 (0.004-0.214) |  |  |
| *24 hours after in µg/kg/min* | 0.063 (0.0185-0.0853) | *0.019* | 0.7422 | 0.265 (0.074-0.548) | *0.165* | 0.1094 |
| *Post administration in µg/kg/min* | 0.074 (0-0.482) | *0.03* | 0.4258 | 0.305 (0.113-0.578) | *0.205* | 0.1602 |

*Abbreviations: Abbreviations: ALAT, Alanine Aminotransferase; AP, Alkaline Phosphatase; ASAT, Aspartate Aminotransferase; CRP, C-Reactive Protein; GGT, Gamma-Glutamyl Transferase; INR, International Normalized Ratio; kU/L, Kilo Units per Liter; mg/dL, Milligrams per Deciliter; U/L, Units per Liter; WBC, White Blood Cells; g/dL, Grams per Deciliter; G/L, Giga per Liter.*

Median (IQR) laboratory and hemodynamic parameters were given for pre-administration, after 24 hours and post-administration. P-values of median differences between pre-administration to 24 hours and pre-administration to post-administration were given aside 24 hours after and post-administration, respectively. Significant median differences and p-values were marked as bold if the p-value was <0.05. Values of Ammonia were lacking in occasional patients. The absolute difference of medians between pre-administration to 24 hours and pre-administration to post-administration are given as “delta Δ” as an italic positive or negative value.

**Supplementary Figures**

**Supplementary Figure S1.**

**
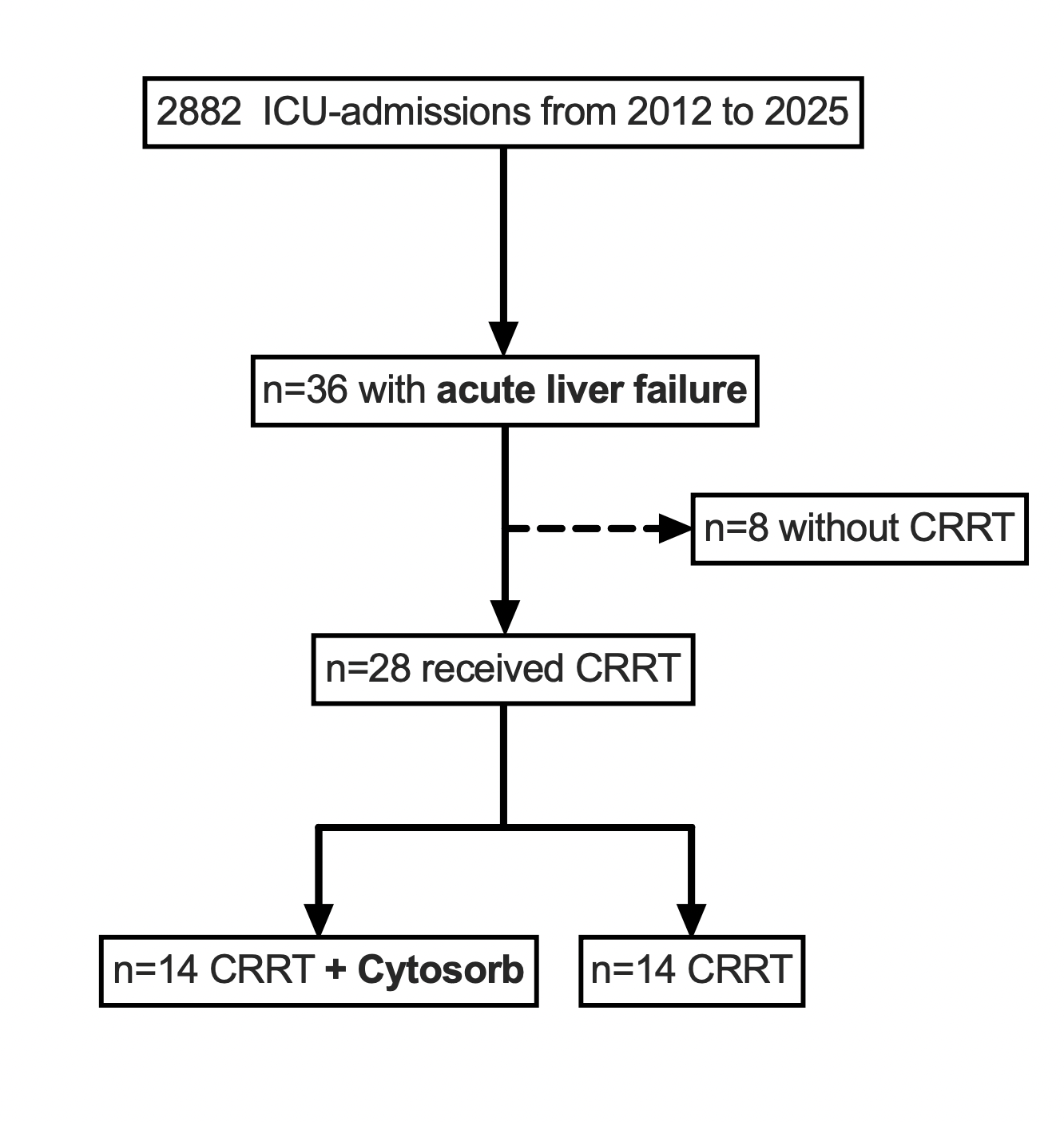
**

**Figure Legend Supplementary Figure S1: Flowchart of the study population.** All patients admitted to the ICU from 2012 to 2025 with Acute Liver Failure were included. However, all patients without continuous renal replacement therapy were excluded from this study population. *Abbreviations: CRRT, Continuous Renal Replacement Therapy; ICU, Intensive Care Unit.*

**Supplementary Figure S2.** Creatinine and BUN.

**Figure Legend Supplementary Figure S2**: **Course of Creatinine and BUN during CRRT and Cytosorb treatment.** Median values of laboratory parameters were given as boxplots with whiskers in the CRRT + Cytosorb group in a light blue box and the CRRT group in a dark blue box. Laboratory values are given pre-treatment, after 24 hours, and post-treatment. Wilcoxon signed rank test was given for median changes in both groups from pre-treatment to after 24 hours or pre-treatment to post-treatment. P-values with p<0.05 were determined as significant and marked with a star. *Abbreviations: CRRT, Continuous Renal Replacement Therapy; BUN, Blood Urea Nitrogen.*

**Supplementary Figure S3.** Lactate and Noradrenaline.

**Figure Legend Supplementary Figure S3:** **Course of Noradrenaline and Lactate levels during CRRT and Cytosorb treatment.** Median values of lactate and noradrenaline were given as boxplots with whiskers in the CRRT + Cytosorb group in a light blue box and the CRRT group in a dark blue box. Whereas lactate levels were available for all patients, we only included patients receiving noradrenaline pre-treatment, after 24 hours, or post-treatment. Laboratory values are given pre-treatment, after 24 hours, and post-treatment. Wilcoxon signed rank test was given for median changes in both groups from pre-treatment to after 24 hours or pre-treatment to post-treatment. P-values with p<0.05 were determined as significant and marked with a star. *Abbreviations: CRRT, Continuous Renal Replacement Therapy.*

**Supplementary Figure S4.** ICU-Survival analysis.

**
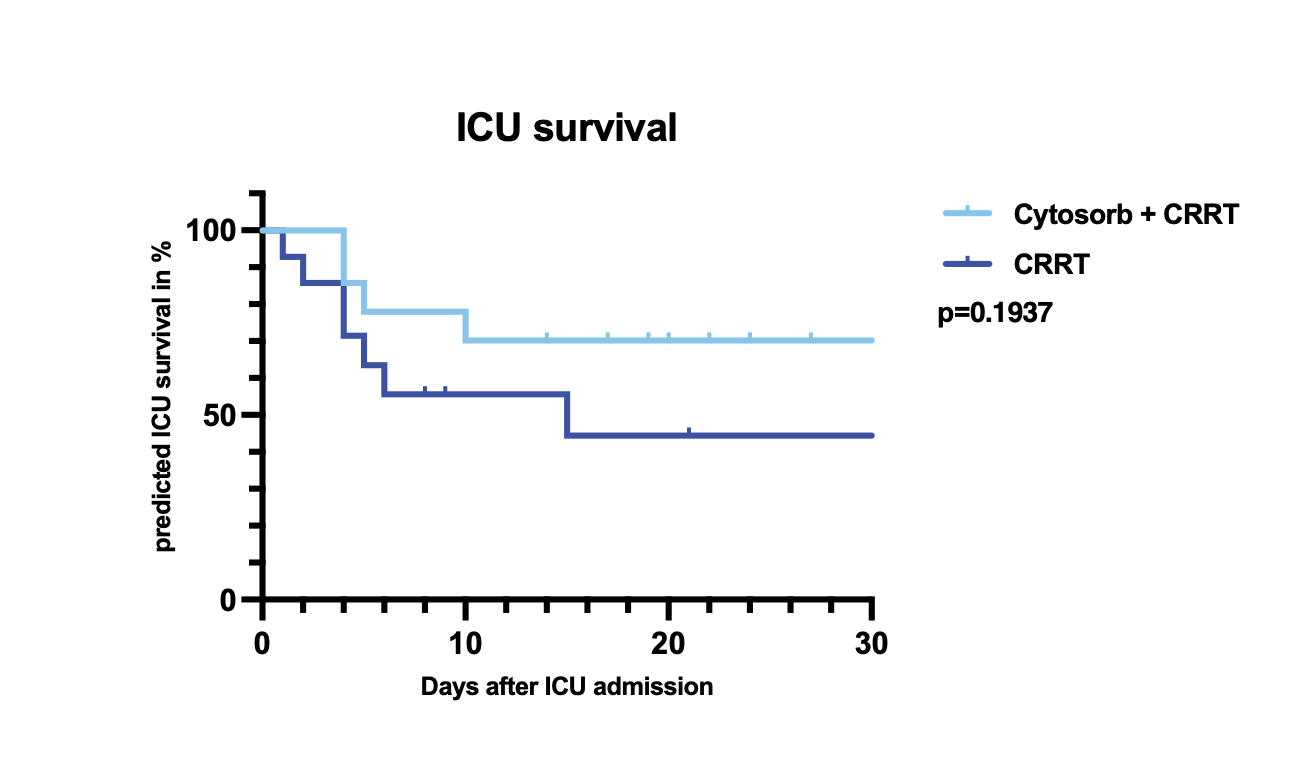
**

**Figure Legend Supplementary Figure S4:** **ICU-Survival analysis.** Patients were separated into a group with Cytosorb+CRRT highlighted as a light blue line and another group with CRRT only highlighted as a dark blue line. Based on a Kaplan-Meier analysis, ICU survival of both groups was calculated, showing a trend toward increased survival in the Cytosorb+CRRT group, but no statistical significance was observed (p=0.1937 by log-rank test). *Abbreviations: CRRT, Continuous Renal Replacement Therapy; ICU, Intensive Care Unit.*
